# Vascular ossification – calcification in metabolic syndrome, type 2 diabetes mellitus, chronic kidney disease, and calciphylaxis – calcific uremic arteriolopathy: the emerging role of sodium thiosulfate

**DOI:** 10.1186/1475-2840-4-4

**Published:** 2005-03-18

**Authors:** Melvin R Hayden, Suresh C Tyagi, Lisa Kolb, James R Sowers, Ramesh Khanna

**Affiliations:** 1 Department of Family and Community Medicine University of Missouri Columbia, Missouri PO BOX 1140 Lk. Rd. 5-87 Camdenton, Missouri 65020 USA; 2 Department of Physiology and Biophysics 500 South Preston Street University of Louisville Louisville, Kentucky 40292 USA; 3 Capital City Medical Associates 1505 Southwest Blvd Jefferson City, Missouri 65109 USA; 4 Department of Internal Medicine University of Missouri School of Medicine Health Sciences Center, MA410, DC043.00 Columbia, Missouri 65212 USA; 5 Department of Internal Medicine University of Missouri School of Medicine Health Sciences Center, MA 436 Columbia, Missouri 65212 USA

**Keywords:** atherosclerosis, atheroscleropathy, HDL-C, lipoproteins, osteoatheroitis, oxidative and redox stress, reactive oxygen species, end stage renal disease

## Abstract

**Background:**

Vascular calcification is associated with metabolic syndrome, diabetes, hypertension, atherosclerosis, chronic kidney disease, and end stage renal disease. Each of the above contributes to an accelerated and premature demise primarily due to cardiovascular disease. The above conditions are associated with multiple metabolic toxicities resulting in an increase in reactive oxygen species to the arterial vessel wall, which results in a response to injury wound healing (remodeling). The endothelium seems to be at the very center of these disease processes, acting as the first line of defense against these multiple metabolic toxicities and the first to encounter their damaging effects to the arterial vessel wall.

**Results:**

The pathobiomolecular mechanisms of vascular calcification are presented in order to provide the clinician – researcher a database of knowledge to assist in the clinical management of these high-risk patients and examine newer therapies. Calciphylaxis is associated with medial arteriolar vascular calcification and results in ischemic subcutaneous necrosis with vulnerable skin ulcerations and high mortality. Recently, this clinical syndrome (once thought to be rare) is presenting with increasing frequency. Consequently, newer therapeutic modalities need to be explored. Intravenous sodium thiosulfate is currently used as an antidote for the treatment of cyanide poisioning and prevention of toxicities of cisplatin cancer therapies. It is used as a food and medicinal preservative and topically used as an antifungal medication.

**Conclusion:**

A discussion of sodium thiosulfate's dual role as a potent antioxidant and chelator of calcium is presented in order to better understand its role as an emerging novel therapy for the clinical syndrome of calciphylaxis and its complications.

## Background

Atherosclerosis and vascular ossification – calcification (VOC) date to the time of the ancient Egyptians [1].

In the initial descriptive phase before the various hypotheses regarding the pathogenesis of atherosclerosis emerged, prominent pathologists of the time emphasized the bone like changes and used the terms ossification and petrification of atherosclerotic lesions.

Benivieni A. in 1507 and Fallopio G. (Fallopius) in 1575 were the first to record for posterity their initial descriptions of atherosclerosis. Fallopius, in 1575, described a degeneration of arteries into bone, which physicians of the time called ossification of the arteries [2]. In 1863, Virchow labeled the vascular changes as "ossification, not mere calcification, occurring by the same mechanism by which an osteophyte forms calcium on the surface of bone" [3]. During the 1800s a widely held concept was that VOC might be a protective mechanism to prevent aortic aneurysms from rupturing. In 1906, Bunting was able to demonstrate the presence of bone marrow containing its classical cellular contents including osteoclasts within a sclerotic aorta. He frequently debated the finding of ossification within the arterial vessel wall (AVW) and supported the finding that ossification was not a rare occurrence; rather it was just not specifically searched for at the time of autopsy [4].

It is currently widely accepted that vascular cells play an active role in osteoid formation and the ossification process to result in VOC. [5-10]

**Figure 1:**
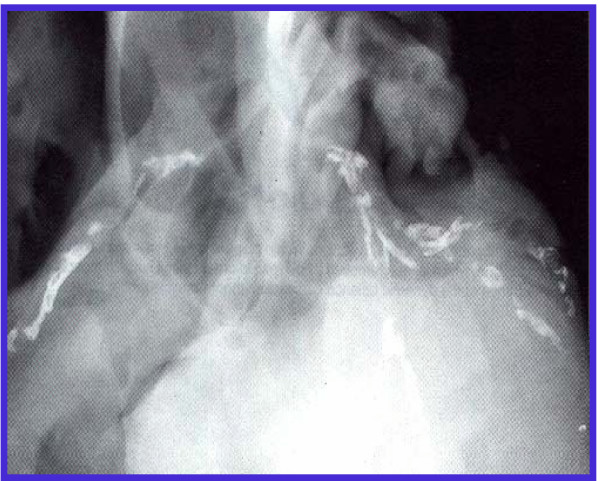
**Coronary artery calcification**. This image demonstrates coronary artery calcification. This 68 year-old Caucasian male with CAD, congestive heart failure and angina was found at autopsy to have a complete occlusion of the LAD with thrombus. Known T2DM of 8 years duration and Htn of 10 years duration with normal CA^++ ^and Pi, BUN 32 mg/dL, creatinine 1.8 mg/dL, proteinuria trace to +1, total cholesterol 198 mg/dL, and triglycerides 398 mg/dL

Newer imaging techniques such as the ultrafast computed tomography, intravascular ultrasound, and emerging data on magnetic resonance imaging are rapidly changing the way we view VOC. These newer techniques (as compared to VOC in radiographs) (figure 1) demonstrate that VOC is not just a degenerative end-stage passive process associated with aging, but a dynamic and regulated event, which serves as a marker and occurs early in the natural history of atherosclerosis [11-13]. Currently, it is felt that coronary artery calcification is not a late, degenerative, end-stage process, but is widespread even in the early, positive, outward remodeling phase and is predictive of cardiac events prior to the development of stenotic lesions [11].

## VASCULAR OSSIFICATION – CALCIFICATION (VOC)

**Figure 2:**
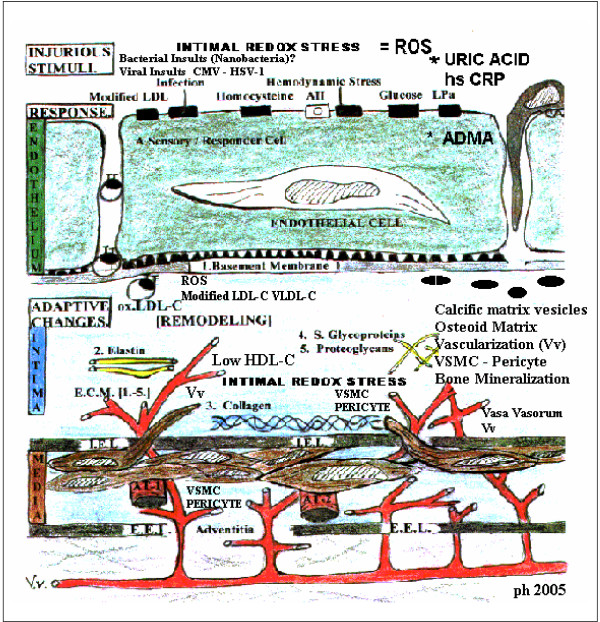
**The central role of the endothelium in VOC and atherosclerosis**. This image portrays the endothelium as the first line of defense against multiple injurious stimuli. Most of the injurious stimuli are represented by the A-FLIGHT-U toxicities found in table 3. When discussing the role of VOC and how it ties into atherosclerosis and the accelerated ASO associated with metS, prediabetes, and overt T2DM it is important to include the various interactions of A-FLIGHT-U toxicities with the associated ROS and the sensitizers for calcium deposition and ossification (**Ca**^++^, **Pi**, **PTH**) within the AVW (both the media and intima) in table 5. The role of ROS, inflammation monocyte-macrophage foam cells undergoing apoptosis – necrosis with creation of a nidus and the stimuli for the VSMC and pericyte following the neovascularization (Vv) of the media and intima to result in osteoid formation and later the mineralization within these atherosclerotic plaques. This image also portrays the possible role for *nanobacteria *in addition to *C. pneumonia *as well as other bacteria and viral infections as possible contributing factors for the ossification process.

**Figure 3:**
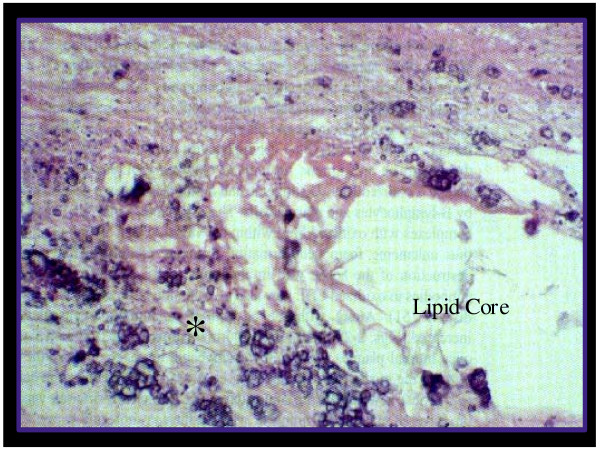
**Matrix vesicle formation at the base of the lipid core**. This image depicts the formation of matrix vesicles at the base of the lipid core in an atherosclerotic plaque. These vesicles are generated through the wound healing process and act as a nidus for later plaque ossification. A 32 year-old male smoker killed in a motorcycle accident. Left anterior descending coronary artery. Note the basophilic staining at the base of the lipid core (starred) and surrounding areas. This area represents early changes of atheromatous ossification. Coronary artery calcification may not be detected until later in life, however this image portrays VOC as being an active directed process beginning early in life.

**Figure 4:**
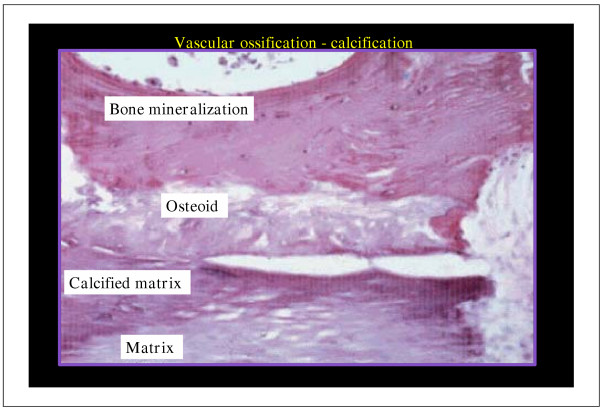
**Transitional stages in VOC**. Transitional stages in vascular calcification recapitulate embryonic endochondral ossification, including an acellular matrix (matrix), amorphous mineralized matrix (calcified matrix), remodeling (osteoid), and following neovascularization of adventitial Vv vessels, complete bone tissue (bone mineralization).

VOC represents a pathologic secondary ossification site or dystrophic calcification primarily in the arterial vasculature in a response to injury mechanism, just as the body responds to the traumatic event of a fractured bone and/or repair of the chondral injury associated with the pathogenesis of osteoarthritis (table 1). In the vasculature this injury is a result of chronic injurious stimuli associated with multiple metabolic toxicities and their associated reactive oxygen species (ROS) generated in the accelerated atherosclerosis (atheroscleropathy) of the metabolic syndrome (metS) and type 2 diabetes mellitus (T2DM), and non-diabetic atherosclerosis (figure 2). There is a recapitulation of embryonic genetic memory associated with this reparative – protective mechanism. In atheroscleropathy and non-diabetic atherosclerosis, the multiple metabolic toxicities and the resultant – parallel ROS, via stimulation of the essential and omnipotent nuclear factor (NFkappaB), which activates the downstream inflammatory mediators TNFalpha, IL-1, IL-6, and other inflammatory markers such as C-reactive protein (CRP). VOC in its early stages develops by a process of matrix vesicle formation usually at the base of the lipid core in the intima of atheromatous plaques (figure 3). These matrix vesicles are then partially replaced with osteoid synthesized by osteoblast-like cells of mesenchymal origin, such as the vascular smooth muscle cell (VSMC) or pericyte. The subsequent bone mineralization is dependent on neovascularization – angiogenesis from the adventitial vasa vasorum (Vv), which leads to formation of mature bone tissue in type Vb atherosclerotic plaques (figure 4) [5,14-16].

This orderly transformation of ossification has been demonstrated to occur in vitro by vascular mesenchymal cells, which undergo a transformation of monolayer growth to swirls, to ridges to a labyrinthine structure [16]

### The vasa vasorum's guardian angel: the pericyte and its important role in VOC

**Figure 5:**
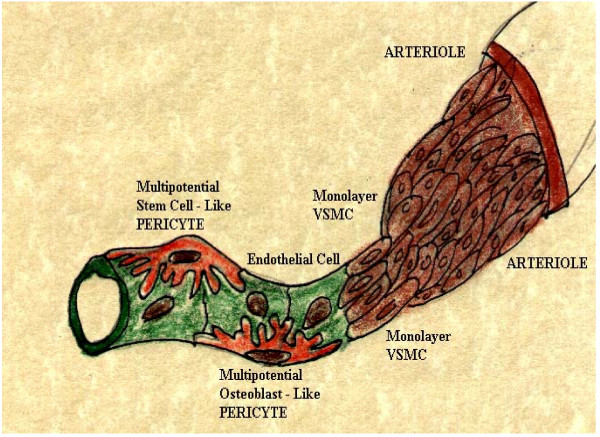
**The multipotential, pluripotent (stem cell – like), and mesenchymal pericyte**. The pericyte is considered to be a differentiated VSMC, which acts as guardian angel of the endothelial cell. It is capable of synthesizing most of the non-collagenous bone morphogenic proteins associated with VOC.

The role of the Vv seems crucial to the development of VOC and its resultant alteration regarding bone formation within the osteoid tissue of the fibrocalcific plaques of atherosclerosis. The medial and intima Vv are derived primarily from the adventitial layer of the artery. The Vv acts as a custom delivery system to supply the media and intimal layers with oxygen and nutrients as the atherosclerotic artery undergoes the positive outward remodeling. These angiogenic vessels also serve as a conduit delivering systemic hormones, cytokines and inflammatory cells to the remodeling media and the atherosclerotic intimal disease of atherosclerosis including ossification. Of particular importance is vital role of the Vv capillary endothelium and its guardian, multipotential, stem cell-like, supportive, mesenchymal cell the pericyte [1,5,17] (table 2) (figure 1, 5). The location of VOC in these arteries is both in the medial and intimal layers. It is important to note that in health the Vv normally penetrates the media but not the intima. In disease the Vv invade excessively not only the media but also the intima and this may help to explain the detrimental process of intimal ossification.

Bone siloprotein synthesized by the osteoblast-like VSMC has recently been shown to be an angiogenic protein capable of inducing neovascularization by interacting with the alpha v beta 3 integrin [18]. Even more exciting is the finding that the endothelial pericyte is capable of synthesizing this non-collagenous angiogenic factor as well as being related to ossification of atherosclerotic plaques.

## VOC IN metS AND T2DM

### Overview

Long before the development of overt T2DM there exists a pro-atherosclerotic process within the arterial vessel wall (AVW) [15,16]. The metS is associated with multiple metabolic toxicities referred to as the A-FLIGHT-U toxicities, which result in the production of ROS (table 3). Each of these excess toxic substrates is capable of generating ROS but in the metS they may act synergistically to result in perturbations of the AVW and contribute to a premature atherosclerosis, which has been termed atheroscleropathy (ASO) [19,20]. This is why many patients with overt T2DM often present with acute coronary syndromes and why T2DM should be considered a coronary heart disease (CHD) risk equivalent according to the National Cholesterol Educational Program Adult Treatment Panel III (NCEP ATP III) guidelines.

### Hyperinsulinemia, Insulin-like growth factor-1 (IGF-1), and Amylin (IAPP)

Insulin, IGF-1, and amylin are known to be elevated in the insulin resistance (IR) and metS patient [21]. Insulin has been long known be a growth factor and is a stimulus for hepatic synthesis of IGF-1, which may be implicated in the accelerated ossification in ASO. There is a human gene polymorphism with a phenotype described as the Benardinelle lipoatrophic syndrome more commonly referred to as lipoatrophic diabetes and this syndrome is associated with manifestations of acanthosis nigricans, generalized lipoatrophy, hirsutism, muscle hypertrophy, intellectual impairment, IR diabetes mellitus with major diet-dependent type V hypertriglyceridemia, and an increase in bone density, thus implying osteogenesis [22].

In addition to insulin and IGF-1, there is another beta cell derived islet hormone that is elevated in IR, metS, prediabetes, and early T2DM termed amylin or islet amyloid polypeptide. Amylin may be considered to be the fraternal twin of insulin and has recently been shown to be a physiologic regulator of bone remodeling through favoring bone formation, while inhibiting bone resorption and thus is osteogenic [23] (table 3).

### Hypertension (Htn)

Htn is the second leading cause of ESRD after diabetes. Hypertension plays an important role in the ASO associated with metS and T2DM, as atherosclerosis is non-existent in the pulmonary arterial tree and the venous system, due to a lower systemic intravascular pressure. Htn may be considered toxic to the AVW and is known to be associated with VOC (table 3) [19,20,24]. Mehrotra R and colleagues have recently demonstrated that hypertension plays a more putative role in the association of coronary artery calcification than do levels of serum calcium, phosphorus, parathyroid hormone, and 1,25 di-hydroxy vitamin D levels in T2DM patients not requiring dialysis [25]

### Hyperlipidemia – Dyslipidemia and the Lipid Triad

The lipid triad of the metS and T2DM consists of an elevation in VLDL-C or triglycerides, small dense atherogenic LDL-C, and a decrease in HDL-C. Once these lipids become modified through oxidation in a milieu of increased ROS from the synergistic effect of the associated A-FLIGHT-U metabolic toxicities, they are a predictive determinate of osteogenesis within the media and intima of the AVW. The earliest changes associated of osteogenesis occur at the base of the lipid core in areas of necrotic-apoptotic macrophages and are associated with ROS and inflamation (figure 3). [1,19,20,26,27].

### Hyperglycemia – Glucotoxicity: Impaired glucose tolerance (IGT) – impaired fasting glucose (IFG) – overt T2DM

**Figure 6:**
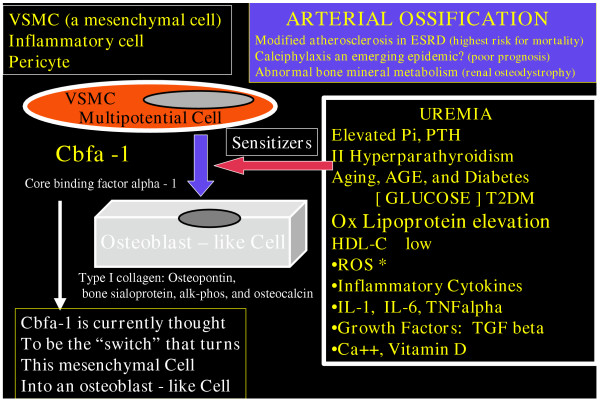
**Sensitizers and the Cbfa-1 "protein switch" to transform mesenchymal cells into osteroblast-like cells**. This slide demonstrates the transformation of the pluripotent mesenchymal VSMC and pericyte into an osteoblast-like cell. It reveals the importance of the ossification sensitizers and core binding factor alpha-1 Cbfa-1 emphasizing their important role in VOC.

Elevated glucose levels (glucotoxicity) have been shown to be a direct sensitizer for osteogenesis [28,29]. In vitro studies with VSMCs and high glucose have been shown to induce cell proliferation and expression of osteopontin, while hypoxia seemed to have an additive effect [28,29]. Even though it is clinically known that diabetes is associated with an increase in medial VOC, the pathogenesis is not completely understood. Glucotoxicity seems to play an important role by transforming VSMCs and possibly pericytes into osteoblast-like cells (figure 6) [19,28,29].

Ishimura E et al have nicely demonstrated the importance of controlling HbA1c levels. They were able to demonstrate that for every 1 percent increase in HbA1c there was a 2.1 fold increase in the risk of VOC [30]. Poor glycemic control seemed to be of greater importance than calcium and phosphate concentrations.

VOC in T2DM involves primarily the media of the AVW, however depending on the degree of underlying ASO; it may frequently involve the intima in those arteries where there is a more extensive involvement of atherosclerosis due to a deeper penetration of the adventitial Vv [1]. Medial artery calcification has been shown to be a strong independent predictor of cardiovascular mortality in patients with newly diagnosed T2DM [31].

In a clinical longitudinal study of 4,553 Pima Indians the risk factors for medial artery calcification in T2DM were impaired vibration perception, long duration of diabetes, and high glucose concentrations as compared to non-diabetic matched controls, whose risk factors for VOC were age, male gender, and high serum cholesterol [32].

### Uric Acid

Serum uric acid (SUA) has long been known for its association with the metS and also it's role in T2DM has recently been discussed in length [33]. SUA is an important substrate in the A-FLIGHT-U multiple metabolic toxicities and contributes to the production of ROS with resultant modification of lipoproteins as well as interfering with the normal function of the eNOS enzyme resulting in an uncoupling phenomenon discussed later (table 3).

Elevated SUA reflects the activity of the xanthine oxidase enzyme and its production of ROS, which subsequently has a positive effect on VOC. Tseng CH has been able to show that SUA is an independent risk factor for the development of peripheral arterial disease in T2DM [34].

### Newer risk factors: Homocysteine (Hcy), inflammation (hs CRP), Microalbuminuria

Each of these novel risk factors plays a significant role in metS – T2DM and contributes to the calcification of the AVW through their association with the multiple metabolic toxicities and production of ROS while contributing to the inflammatory process (table 3).

Hcy is known to be toxic to the AVW resulting in a host of fibrotic remodeling changes as a result of autoxidation, VSMC activation, and proliferation, as well as the production of ROS [35]. Li J. et al. have been recently able to demonstrate that Hcy potentiates calcification of cultured rat VSMCs [36].

Mediators of inflammation such as oxidation, carbonyl stress, C-reactive protein (CRP), and cytokines may directly stimulate vascular calcification. Also, inflammation reduces fetuin-A, a naturally occurring inhibitor of vascular calcification, which binds excess mineral in serum [37].

Increased levels of CRP are significantly associated with the presence of vascular calcification (including atheromatous and medial calcification) in both aorta and hand arteries, which may indicate a relationship between inflammation and vascular calcification in hemodialysis patients [38]. It is of interest to note that IL-10 plays an important role as an anti-inflammatory cytokine and is known to be decreased in CKD and ESRD. Low monocyte IL-10 synthesis may contribute to a chronic inflammatory state in these patients by defective feedback inhibition of proinflammatory cytokine production [39]. While inflammation plays a very important role, it is important to note that ROS occurs upstream from inflammation and that ROS are an important mediator of the nuclear factor kappa B [33,34].

Microalbuminuria is recognized as a surrogate marker for systemic endothelial dysfunction [40]. Recently, microalbuminuria has been shown to be a strong predicting factor of medial arterial calcification independent of nephropathy in patients with diabetes [41].

## VOC IN CHRONIC KIDNEY DISEASE (CKD)

Largely due to the prevalence of T2DM and Htn, approximately 11% of the U.S. population has some manifestation of CKD. This spans the proteinuria phase with normal renal function to advanced ESRD requiring renal replacement therapy either by dialysis or transplantation [42]. To better grasp this effect of CKD, the USRDS 2004 annual report has estimated there are in excess of 400,000 people undergoing some form of renal replacement therapy in the U.S. [43]. CKD should be considered in the highest-risk category for the development of cardiovascular disease (CVD) and an aggressive team approach of global risk reduction in order to reduce the prevalence and severity of the associated cardiovascular events should be established by following the RAAS acronym (table 4).

CVD accounts for the largest cause of death in ESRD patients and may range from 10 – 30 fold higher for those patients being treated with dialysis as compared to the general population [44]. Patients entering dialysis over the past two decades now have a much higher incidence of diabetes and this is up to 59% as a first or second diagnosis with hypertension being the second leading cause of ESRD. Higher Framingham risk scoresand an aging population at the time of entry as a result of current technology and newer medications are preventing earlier coronary events and allowing them to survive and develop other end organ disease, such as ESRD and congestive heart failure, which are partly responsible for the increasing number of these patients [45].

Is atherosclerosis accelerated in this patient population or is there some other factor(s) related to the cardiovascular toxicity associated with uremia and dialysis?

Schwarz U et al. were able to demonstrate an increased size of the medial layer, marked increased calcification, increased number of calcified plaques, and a decreased lumen area in a study of 27 patients with ESRD compared to 27 non-renal disease controls that had died due to coronary atherosclerosis. Thus, there was no evidence to suggest an accelerated atherosclerosis, but instead, a major change in plaque composition with an increase in ossification and medial enlargement [46].

Just as there are chronic injurious stimuli in atherosclerosis and ASO the pathology of CKD and ESRD include additional injurious stimuli to the vascular cells of the AVW. These include an elevation of the following: Phosphorus (Pi), calcium (Ca^++^), parathyroid hormone (PTH), hypoxia and resultant ROS formation, and others (table 5) (figure 6). VOC is the manifestation of a complicated systemic process that begins primarily with medial ossification of larger and medium size arteries and progresses to the smaller arterioles. Concurrently, there is a development of renal osteodystrophy and ossification in the surrounding articulations of multiple joints [osteoatheroitis – osteoarthritis – renal osteodystropthy (table 1)]. The clinical manifestations of VOC depend on the location of the affected arteries and arterioles [47-50].

### Atherosclerotic nephropathy

Scoble JE [51] presented the term atherosclerotic nephropathy in 1999, which includes abdominal atherosclerosis, renal artery atherosclerosis with renal artery stenosis, and atheroembolic disease. In older age populations, it has been estimated that atherosclerotic renal artery disease accounts for 14% (over the age of 50) and 25% of patients (over the age of 60) with end-stage renal failure [51,52]. With the age groups over the age of 50 and 60 (baby boom – senior boom generation) expanding at an exponential rate in western countries we will see an increasing number of patients with ESRD attributable to this abnormality.

This atheroembolic phenomenon accounts for untold cases of ischemic nephropathy with functional loss of nephron units. Associated clinical companions of this condition that might alert the clinician would be: rapid increasing proteinuria, rapid deterioration of renal function, and other clinical evidence of peripheral atheroembolic phenomenon in other vascular beds such as the extremities in peripheral vascular disease (blue toe syndrome), mesenteric thrombosis and associated gastrointestinal bleeding, and extra cranial cerebrovascular atherosclerosis with associated transient ischemic episodes or overt cerebral thrombosis.

In atherosclerotic coronary artery disease we do not see evidence of intra-myocardial atherosclerosis and likewise, we do not find evidence of intrarenal atherosclerosis. The renal artery may be likened to an epicardial coronary artery when comparing these two diseases. It is currently accepted that atherosclerotic coronary artery disease and hypertension account for the major cause of death and congestive heart failure in developed countries. It is time to consider atherosclerotic renal artery disease with VOC and associated renal artery stenosis, as well as, hypertension as a significant cause of progressive CKD and its progression to ESRD.

As present in any atherosclerotic lesion, VOC is known to occur and will increase in the patient with CKD and ESRD (especially the patients with diabetes and ASO). VOC detected by non-invasive spiral CT scanning would alert the clinician to the possibility of renal artery stenosis and additionally alert the clinician to the importance for more aggressive therapeutic measures to control the A-FLIGHT-U toxicities (table 3) as well as to control the metabolic toxicities of the increased known sensitizers to VOC (table 5) (figure 6).

## VOC IN CALCIPHYLAXIS (CPLX) – CALCIFIC UREMIC ARTERIOLOPATHY (CUA)

Any discussion of VOC would be incomplete without discussing the important issue of CPLX – CUA and a better understanding of this condition merits a janus-faced approach. The century old CPLX – CUA clinical syndrome, which once was considered to be a rare clinical phenomenon primarily associated with CKD and ESRD is now being reported and being seen in increasing numbers. Part of this increase may well be due to a better understanding and recognition of this syndrome and previous underreporting; however nephrology and dermatology departments, and dialysis clinics throughout the U.S. are seeing increasing numbers of this morbid – mortal complicated clinical syndrome. As the number of patients with CKD and ESRD continue to grow in numbers the medical community may well see this syndrome in near epidemic proportions (especially considering the current obesity – metS – T2DM epidemic).

CPLX is not unique to any one country and is a global phenomenon. As with any new and emerging clinical syndrome the literature is replete with multiple case reports of this entity. In this review, an attempt is made to increase the awareness and understanding of this malady and provide an overview and not reference the multiple rare and unusual case reports, unless they contribute to the understanding of mechanisms or treatment [53-63].

### Historical perspective of CPLX

Selye's initial experiments in rats set the stage for the description of this clinical entity in humans [64]. He hypothesized that sensitizers such as PTH or vitamin D might condition the vasculature to VOC. After providing the sensitization he then challenged the rats with known provoking factors including metal salts, albumin, and trauma, which induced calcium deposition and subsequent inflammatory necrosis of the skin and subcutaneous tissue. Over time other studies suggested alternate contributing factors, such as those listed in table 5. The name originated from the combination of calcium and anaphylaxis, thus calciphylaxis, however this model does not involve IgE mechanisms.

Byrant and White (1898) are credited as having described the first clinical presentation in a six-month-old infant, in which there was VOC and what they termed endarteritis obliterans, indicating involvement of the intima [65]. It was not until 1969 that Rees and Coles used the terminology calciphylaxis to describe this clinical and pathologic syndrome in man [66].

Coates (1998) coined the term calcific uremic arteriolopathy (CUA) as a replacement term for CPLX, since in many instances elevations of Ca++, Pi or PTH levels are absent [67]. Over time CUA may become the preferred term, as this newer terminology is only six years old. The term calciphylaxis is almost entrenched in the medical literature at this point in time; however Selye's animal model more closely parallels the dystrophic tumorous soft tissue calcifications of uremic CPLX. His model did not exhibit the histology consistently described with uremic CPLX, i.e. small vessel calcifications and intimal hypertrophy in association with panniculitis (lobular fat necrosis, and inflammatory changes with neurtrophils, lymphocytes and macrophages, and small vessel thrombosis or involve IgE mechanisms [54,67-69]. We favor the term: obliterative calcific vasculopathy.

**Figure 7:**
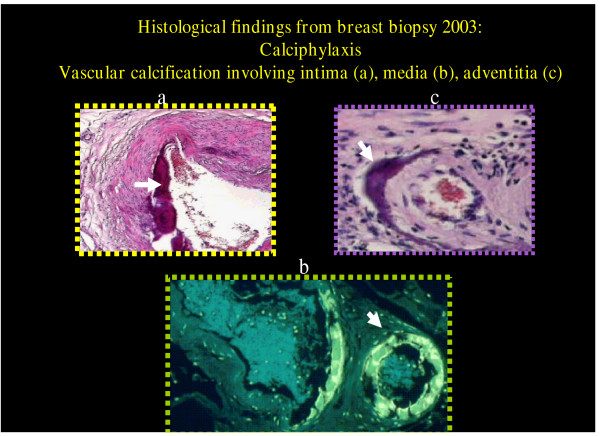
**Histologic changes in a patient with CPLX and ESRD**. Breast biopsy (benign) from a non-diabetic 60-year-old Caucasian female with an irregular breast mass 12 months prior to the development of clinical abdominal CPLX (multiple tumorous calcifications in the abdominal adipose tissue and skin ulceration). These tissue sections demonstrate the underlying systemic VOC of calciphylaxis. Her ulcerated area on the abdomen was not biopsied due to a possibility of aggravating tissue healing. Panel a: Demonstrates the H&E basophilic staining of intimal VOC in a small musculoelastic artery. Panel b: Demonstrates the medial fluorescent-like staining (due to inverted coloration of H & E basophilic staining) of VOC in an arteriole. Note the adjacent venule medial VOC staining. Venular VOC – thrombosis has not been as extensively studied as the arteriole and one must consider the possibility that VOC – thrombosis in the post-capillary venule may be important as the increased capillary edema and pressure may result in capillary endothelial dysfunction and promote an additive factor in the important role of subcutaneous ischemia and skin necrosis and ulceration. Note the panarteriolar involvement of the intima, media, and adventitia in the various vessels. Panel c: Demonstrates the H & E basophilic adventitial staining of VOC in an arteriole.

VOC in the syndrome of CPLX – CUA is an important topic for discussion, as it is being reported at an exponential rate and especially over the past two years. The devastating complication of medial vascular calcification of small arterioles with resulting skin necrosis is rapidly becoming a morbid complication in both the non-diabetic and especially the large numbers of diabetic patients (T2DM > T1DM) undergoing renal replacement therapy (figure 7).

### The skin in CPLX

The skin lesions of CPLX present as painful violacous (livedo reticularis) areas in the extremities, proximal medial thighs, anterior abdomen and lateral flank areas, the breast in females, and frequently overlie or are adjacent to subcutaneous tumorous calcifications in areas of increased adipose tissue. They then progress to a blackened eschar, which later reveals itself on a granulating bed and enlarges at the periphery of the ulcer. They have a tendency to develop in sites of prior skin trauma, such as abdominal scars from previous surgical incisions and may even develop from the trauma of simple scratching of extremities due to ESRD-associated pruritis.

It is important to recognize the clinical presentation of the skin color changes and the neuropathic type of pain before ulceration has developed. If we wait until skin ulceration develops we place the CPLX patient at an increased risk of sepsis and premature death. We should carefully assess any palpable calcific tumorous masses in the subcutaneous tissue in patients at high risk for CPLX.

Not all skin ulcerations are due to CPLX and therefore we should utilize our differential diagnostic skills in evaluating ulcers in these high-risk patients with CKD, T2DM, and ESRD. A reasonable differential diagnosis might include the following (table 6). Even though a skin biopsy may show medial arteriolar calcification and atrophy of medial muscle layers in the arteriole, we should keep in mind that these changes although quite specific are not pathognomonic for CPLX.

The recent emergence of a newer skin pathology termed: neprogenic fibrosing dermopathy may appear co-morbidly with CPLX and recently it has been reported that transforming growth factor beta-1 (TGF beta-1) may to be related to both disorders [55]. By making an accurate and earlier diagnosis of CPLX, a greater effort may be given by the primary care provider and the nephrology team as well as other specialties (dermatology and plastic surgery) to improve the multiple A-FLIGHT-U toxicities in each patient (table 3). Earlier treatment of each altered abnormality of the associated multiple metabolic derangements may prevent or delay the progression of this morbid mortal clinical syndrome (table 4,5).

### Endotheliopathy, redox stress, and reactive oxygen species. eNOS and eNO

**Figure 8:**
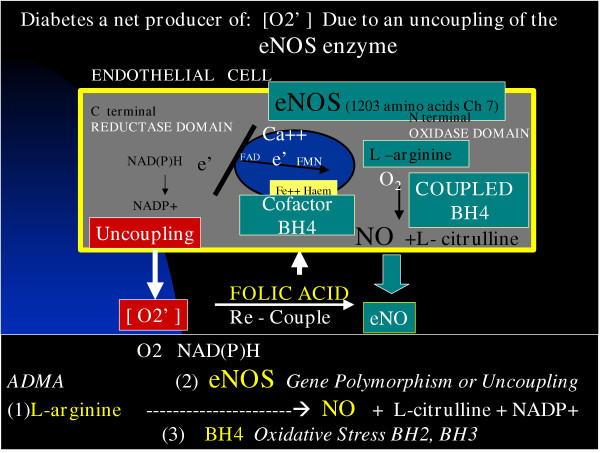
**Uncoupling of the eNOS enzyme resulting in the net production of superoxide instead of the protective endothelial nitric oxide (eNO)**. This image depicts the eNOS enzyme reaction and resultant production of the protective antioxidant, anti-inflammatory gas product eNO. Oxygen reacts with the eNOS enzyme in which the tetrahydrobiopertin (BH_4_) cofactor has coupled nicotinamide dinucleotide phosphate reduced (NAD(P)H) enzyme with L-arginine to be converted to nitric oxide (NO) and L-citrulline. When eNOS uncoupling occurs the NAD(P)H enzyme reacts with O_2 _and the endothelial cell becomes a net producer of superoxide (O_2_^•^) instead of the protective endothelial NO. This figure demonstrates the additional redox stress placed upon the arterial vessel wall and capillaries in patients with MS, PD, and overt T2DM.

The endothelium is an elegant symphony responsible for the synthesis and secretion of several biologically active molecules. It is responsible for regulation of vascular tone, inflammation, lipid metabolism, vessel growth (angiogenesis – arteriogenesis), arterial vessel wall – capillary sub endothelial matrix remodeling, and modulation of coagulation and fibrinolysis. One particular enzyme system seems to act as the maestro: The endothelial nitric oxide synthase (eNOS) enzyme and its omnipotent metabolic product: endothelial nitric oxide (eNO) (figure 2,8).

The eNOS enzyme reaction is of utmost importance to the normal functioning of the endothelial cell and the AVW. When this enzyme system uncouples, the endothelium becomes a net producer of superoxide and ROS instead of the net production of the protective antioxidant properties of endothelial derived nitric oxide (table 7) (figure 8). There are multiple causes for eNOS enzyme uncoupling: The A-FLIGHT -U multiple metabolic toxicities, oxidative – redox stress, ROS, metS, insulin resistance, prediabetes, T2DM and T1DM, Htn, endothelin, and asymmetrical dimethyl arginine (ADMA) [1,19,20,27,28,34,45].

The endothelial cell is important in controlling the function of small arterioles and how they may become a net producer of ROS. This endothelial dysfunction may precede the vascular narrowing – closure, prothrombotic state, thrombosis with resulting ischemia of the surrounding subcapillary interstitium, ischemic necrosis of the subcutaneous adipose tissue, and skin changes of livedo reticularis with transformation to painful necrosis, eschar formation, and skin ulceration.

In addition to correcting the underlying multiple metabolic A-FLIGHT toxicities and positive regulators of VOC (table 3,5), a strong consideration to newer therapies should be entertained in order to prevent, slow, and alter the morbid – mortal natural history of the dreaded complications associated with CPLX – CUA.

## Future Directions

### Sodium thioSulfate (STS) intravenous therapy for calciphylaxis: its emerging role

**Figure 9:**
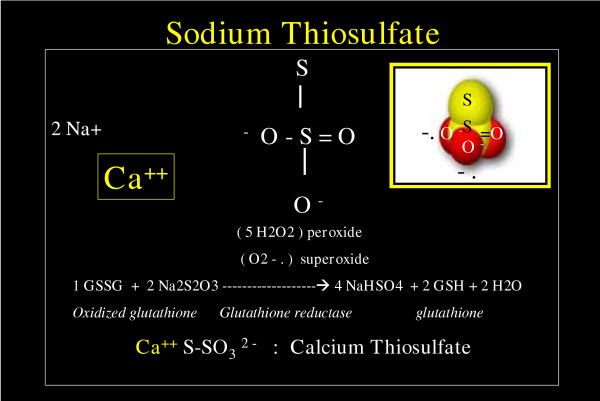
**Sodium thiosulfate**. This figure demonstrates the chemical structure of sodium thiosulfate, which is an antibrowning, reducing, and antioxidant agent: Capable of donating electrons to re-pair unpaired damaging electrons to be an effective antioxidant as well as a chelator of cations such as the calcium excess in calcific uremic arteriolopathy – CPLX.

STS is an effective antibrowning, reducing, and antioxidant agent and therefore readily donates electrons to re-pair unpaired damaging electrons to be an effective antioxidant in addition to it use as a chelator of cations such as the calcium excess in CPLX – CUA (figure 9). The following is one of many possible antioxidant reactions as it scavenges ROS and may be responsible for producing glutathione:



STS is known to be a chelator of cations and has been used for over a century as an antidote for cyanide toxicity, topical treatments of acne and versicolor, and recently as a chemoprotectant against carboplatin and cisplatin toxicity. Its use in the treatment of calciphylaxis has only recently been described and its future use may become a standard of care in the treatment of the dreaded complications [70]. Its dual role as an antioxidant and chelator makes STS an excellent choice for the treatment of calciphylaxis and it will be interesting to follow future clinical trials regarding it use.

Clinically, STS seems to have a rather intriguing rapid response in the relief of the pain associated with CLPX and it frequently reduces pain within the very first few treatments (personal experience). Additionally, it seems to promote healing of skin ulcerations especially when used in conjunction with hyperbaeric oxygen treatments [70].

### The STS hypothesis

STS may restore endothelial cell dysfunction in CPLX through its antioxidant actions and have a positive effect on eNOS uncoupling and eNO generation. We have formed a hypothesis that the rapid reduction in pain may be due to a restoration of endothelial dysfunction associated with this syndrome. The anitoxidant effect of STS, given in the intravenous dosage of 12.5–25 grams intravenous at the end of dialysis may help to restore the dysfunctional endothelial cell and begin restoring the endothelium's natural tendency (in health) to produce eNO (promoting vasodilation) instead of the dysfunctional super oxide and the resultant peroxynitrite (promoting vasoconstriction) (figure 8) [1,19,20,27,28,34,45].

**Figure 10:**
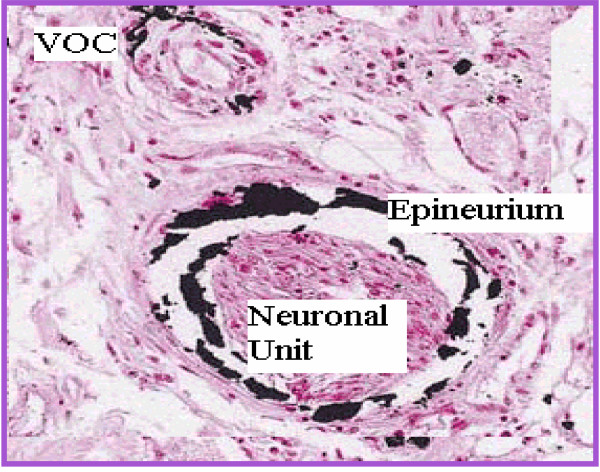
**Neuronal calciphylaxis**. This image demonstrates not only medial calcification of an arteriole but also calcification of the epineurium of a peripheral neuronal unit within the subcutaneous tissue of a patient with systemic CPLX. This is from the same patient and breast biopsy tissue as in figure 7. Calcium stains black in this von Kossa stain.

STS could be working in two different vascular beds in the vulnerable subcutaneous tissue, as the peripheral neuronal unit is also involved with dystrophic ossification – calcification (figure 10): The vasa nervorum and the endoneurium of the peripheral neuronal unit [71] and the distal small pre-capillary arterioles. These changes would help to explain the rapid improvement of this morbid neuritic (breathtaking, stinging, burning, and knife-like) type of pain, as the chelation properties of STS would take much longer (months compared to hours or days) to restore the capabilities of the neuro-circulatory vascular beds.

Diabetic and peripheral arterial disease patients may be slower to respond as they are plagued with a much more intense endothelial cell dysfunction and endothelial nitric oxide enzyme uncoupling due to the advanced atherosclerotic process and medial calcifications and associated ischemic changes.

Undoubtedly the essential cofactor tetrahydrobiopterin (BH_4_) becomes depleted during this chronic ischemia in the subcutaneous tissue from the multiple metabolic toxicities. STS may have a "folate-like redox shuttle effect" similar to the pleiotropic effects of folic acid supplementation [34] by allowing the oxidized cofactor BH_3 _and BH_2 _to undergo a restoration by the antioxidant effects of STS to the requisite completely reduced form BH_4_. This may ameliorate the underlying oxidative stress within the pre-capillary and capillary beds of the interstitium and the endoneurium of the peripheral neuronal units involved. While this is only a hypothesis it seems to be clinically relevant due to the clinical observation of a rapid relief of the pain associated with CPLX.

### Emerging role of nanobacteria

Currently, the multiple roles of nanotechnology and nanobacteria are rapidly emerging and with new findings come new ideas, which may have a significant impact regarding the future of medicine. Nanobacteria may be a causative agent of diseases related to the biomineralization processes. As clinicians and scientists we must be careful as newer hypothesis and treatments emerge. For example even though nanobacteria have been found in multiple disease states associated with calcium deposition, we have yet to fulfill Koch's postulates regarding VOC. Because this information has just evolved it is important to review some of the background in this field of study.

We have witnessed the exciting story of *H. pylori *and its causative role in ulcer disease and followed the *C. pneumonia *story in atherosclerosis finding it to be one of the burdensome infectious injurious stimuli to the endothelium that results in remodeling and atheromatous changes in the AVW. Likewise, the new information regarding nanobacteria being associated with VOC is exciting and could play a very important role in the causation of the atheromatous process and VOC (figure 2).

Nanobacteria belong to the family of gram-negative rods (approximately 100 fold smaller in size) and have been recently localized in cardiovascular tissue. Miller VM et al. have found nanometer-scale particles (ranging in size from 30 to 100 nm) similar to those described as nanobacteria isolated from geological specimens and human kidney stones. These ultra small microbes can be visualized in and stained immunohistologically from calcified human cardiovascular tissue [72]. Jelic et al. have demonstrated the presence of nanobacteria in a mitral valve associated with a localized calciphylaxis within mitral vegetations from a patient with T2DM [73].

Maniscalco BS et al., have been able to demonstrate a statistically significant improvement in coronary calcium scores (inferring a reversal of VOC) and a reduction in angina in a group of patients with CAD and coronary artery calcifications using thecombination of EDTA by rectal suppository and a combination of multiple known antioxidants with 500 mg tetracycline (nanobacteriocidal) given daily by mouth. The treatment duration was 4 months [74].

Recently the literature has been replete with the association of periodontal disease and its relation to cardiovascular disease and events. Whether this condition is responsible for the activation of the chronic inflammatory disease response driving the atherosclerotic process or not remains to be seen. The recent isolation of nanobacterium with periodontal disease has fueled the fire of this being a putative microorganism in the development and progression of atherosclerotic disease and VOC [75].

### Osteoporosis and VOC

The coexistence of osteoporosis, CVD, and VOC with the aging process in humans and the concept of a shift of calcium from the osseous skeleton towards the AVW have been suggested as one of the explanations for their simultaneous existence [76,77].

Accumulating evidence indicates that there are similar pathophysiological mechanisms underlying both diseases. The A-FLIGHT-U multiple metabolic toxicities and resultant ROS, oxidative stress, endothelial cell dysfunction, endotheliopathy, and inflammation have been central issues in this paper regarding VOC. Each of these is associated with a modulation of vascular and osteogenic cells but in an opposite manner and may act in a synergistic manner. The finding of oxidative stress promoting VOC while promoting osteoporosis may help to explain the paradoxical parallel buildup of VOC and the concurrent loss of bone mineralization in the osseous skeleton [78-80]. The current evidence linking both of these diseases is far from conclusive and additional research is necessary to further characterize the relationship between these two common illnesses as our population of baby (senior) boomers are aging in large numbers.

One such exciting and current emerging area in vascular biology involves the RANKL/RANK/OPG system, which belong to the tumor necrosis factor-related family recently discovered to be critical regulators of the immune system and skeletal biology. RANK(L) (receptor activator of nuclear factor kappaB (ligand) may promote and osteoprotegerin (OPG) may protect against vascular calcification (table 5). The mouse OPG knockout model displays osteroporosis and increased vascular calcification and human gene polymorphisms of OPG are currently being defined and how they may confer an increased risk of CAD in Caucasian men while interacting with VOC and osteoporosis [81]. The finding that OPG levels may reflect endothelial cell dysfunction is certainly an area under serious consideration [76,82].

### CPLX and T2DM

The diabetic patient is already plagued with an underlying accelerated atherosclerosis (atheroscleropathy (ASO)), as well as, medial fibrosis and VOC (even if they do not develop CKD or ESRD requiring dialysis). With the current epidemic of obesity, metS, insulin resistance related T2DM (diabesity) in all age groups and particularly the aged and adolescent youth, a closer examination of this morbid – mortal complication is in order [83-87].

If these T2DM patients do develop CKD and/or ESRD requiring dialysis they could be at a much higher risk of developing an even more severe form of VOC. This dual effect of having not only T2DM related medial calcification but also having the increased risk for the development of medial remodeling and VOC from CKD and/or ESRD requiring dialysis would place these individuals at an extremely high risk for the development of excessive medial arterial remodeling and VOC, as well as arteriole remodeling and VOC, which could possibly increase the risk of developing CPLX (figure 2, 7). Many T2DM patients may have medial arteriolar calcification that could be contributing to anunderlying skin necrosis currently being attributed singularly to peripheral arterial disease and ischemic necrosis or gangrene, when if fact they might have coexisting arteriolar calcification contributing to their skin changes of gangrene. This dual mechanism of VOC in the arterioles and conduit arteries could be playing an important role in development of CPLX.

The treatments to date have largely consisted of careful wound management, hyperbaric oxygen therapy [88], and specific treatment of wound infections, which frequently become systemic and result in deadly sepsis, organ failure, and patient demise. These open ulcerations of the skin are extremely difficult to heal and while they remain open they place the patient at an extreme risk of infection, as this patient population is known to have an impaired immune system from the combination of dialysis and T2DM [89]. If STS does emerge as a treatment option for those with CPLX then a careful evaluation of the T2DM patient with skin ulceration should be explored and consideration given to the use of STS provided there is evidence of an associated arteriolar calcification in addition too medial calcification of the conduit arteries. The dual effect of STS being an antioxidant and a chelator of calcium could have a positive outcome in promoting healing of skin ulcerations, thus reducing the possibility of systemic infection and furthermore result in limb salvage of these high risk patients who seem to be presenting to dialysis clinics more and more as the obesity – T2DM epidemic continues to progress. Additionally, the use of STS may be considered in the treatment of VOC associated with CHD and peripheral arterial disease independent of CKD and dialysis.

## Conclusion

This review has been an attempt to increase our basic understanding of VOC and its mechanisms in the patient with metS, T2DM, CKD, CHD, and the syndrome of CPLX. By improving our understanding of the mechanisms in play we may be better equipped to prevent many of the complications associated with VOC.

VOC is an exciting and complicated field of study and while our understanding of this process has undergone considerable improvement regarding its mechanisms and understanding during the past few years, there remain many questions to be solved and newer therapies proposed to help decrease the morbid – mortal complications of this actively regulated process.

The recent publications of case reports regarding the use of STS to treat the complications of VOC and the syndrome of CPLX may result in the necessary clinical trials and the important scientific animal experiments to fully evaluate and understand its effect. Additionally these early published clinical results should instigate the necessary studies to better understand the mechanisms of this emerging treatment for calciphylaxis and its possible role in treating VOC in patients with CHD, atherosclerosis and atheroscleropathy in metS, and T2DM. Additionally, this emerging treatment may have significant applications for diabetic patients with CKD, ESRD. and VOC.

Earlier diagnosis of the patient at risk for the development of VOC is important so that a global risk reduction program can be initiated in order to control for the multiple metabolic toxicities by applying the therapeutic RAAS acronym (table 4) for the treatment and prevention of complications [90]. While this remains a daunting task for clinicians it can be undertaken by a team effort approach involving our multiple resources and sub-specialties in medicine.

## Competing Interests

The author(s) declare that they have no competing interests.

## Authors' Contributions

MRH conceived the idea to write this manuscript. MRH, SCT, LGK, JRS, and RK wrote, and edited this manuscript jointly.

## List Of Abbreviations

ASO – atheroscleropathy

AVW – arterial vessel wall

CHD – coronary heart disease

CKD – chronic kidney disease

CPLX – calciphylaxis

CRP – C-reactive protein

CUA – calcific uremic arteriolopathy

CVD – cardiovascular disease

eNO – endothelial nitric oxide

eNOS – endothelial nitric oxide synthase

ESRD – end stage renal disease

GLA – gammacarboxyglutamate

Hcy – homocysteine

HDL-C – high density lipoprotein cholesterol

hsCRP – highly sensitive C-reactive protein

Htn – hypertension

IGF-1 – insulin like growth factor-1

IL-6(10) – interleukin 6 and 10

LDL-C – low density lipoprotein cholesterol

metS – metabolic syndrome

MGP – Matrix gammacarboxyglutamate (Gla)-protein

NFkappaB – nuclear factor kappa B

OPG – osteoprotegerin

PTH – parathyroid hormone

RANK – receptor activator of nuclear factor kappaB

RANKL – receptor activator of nuclear factor kappaB ligand

ROS – reactive oxygen species

SUA – serum uric acid

TC – total cholesterol

T1DM T2DM – type (1) (2) diabetes mellitus

VLDL-C – very low density lipoprotein cholesterol

VOC – vascular ossification – calcification

VSMC – vascular smooth muscle cell

Vv – vasa vasorum

## Figures and Tables

**Table 1:** Tissue wound injury: a common link between two chronic diseases: OSTEOATHEROITIS and OSTEOARTHRITIS.

**T**	**Tissue wound healing: a recapitulation of embryologic genetic memory**
**I**	**Injury – Inflammation: OSTEOATHEROITIS – OSTEOARTHRITIS**
**G**	**Granulation tissue formation (angiogenesis)**
**E**	**Endothelial cell activation, proliferation, migration: ANGIOGENESIS**
**R**	**Remodeling of the AVW: **initially positive outward remodeling and over time a chronically negative inward arterial remodeling (see Scar contracture). **Repair.** **Restructure.** **Resolution**: results, only if the chronic injurious stimuli and sensitizers are removed from the endothelial, intimal, and medial layers of the arterial vessel wall. **The above 4 (R's): **activate pleuripotent mesenchymal stem-cell pericytes and VSMCs to produce bone morphogenic proteins, which are also activated by (I): Injury and Inflammation with resulting inflammatory cytokines such as TNF alpha, IL-6 and decreased IL-10.
**S**	**Scar**: contracture and negative remodeling resulting in stenosis. AVW stiffness even without ossification.

**Table 2:** Vasa vasorum: a custom delivery system

1.	Supply the media and intima with vascular mesenchymal cells:
	a. The multipotential, stem cell-like cell to contribute to the substrate of cells to produce an osteoid matrix by morphogenic bone proteins
	b. A custom delivery system for Pi, Ca^++^, Glucose, Insulin, IGF-1, PTH, and other activators of bone formation
2.	Substrates of the Renin Angiotensin Aldosterone System.
3.	Substrates of native LDL-cholesterol and modified LDL-cholesterol. Substrates of phospholipids from systemic circulating cells.
4.	Substrates for angiogenesis. The endothelial progenitor cells, as well as, the endothelial cell and the pericyte itself via migration.
5.	Supply the route for the "second wave" of inflammatory cells to the intima and vulnerable shoulder regions of the plaque, fueling the inflammatory process. The first wave originating from the endothelial luminal surface.
6.	Supply the direct route for adventitial fibroblasts and TGF-beta allowing for intimal plaque expansion.

**Table 3:** The multiple metabolic toxicities of the A-FLIGHT-U acronym

**Multiple injurious stimuli responsible for the production of ROS.**	
**A**	Angiotensin II (also induces protein kinase C – β isoform) Amylin (hyperamylinemia) islet amyloid polypeptide toxicity AGEs/AFEs (advanced glycosylation/fructosylation endproducts) Apolipoprotein B Antioxidant reserve compromised Absence of antioxidant network Aging ADMA (Asymmetrical DiMethyl Arginine) Adipose toxicity: **Obesity toxicity – Lipid Triad **(decreased HDL-C, increased triglycerides and small dense LDL-C) Adipocytokine toxicity: Increased TNF alpha, Il-6, TGF beta, PAI-I and the increased hormones resistin, leptin and decreased adiponectin.
**F**	Free fatty acid toxicity: **Obesity toxicity – Lipid Triad**
**L**	Lipotoxicity – Hyperlipidemia – **Obesity toxicity – Lipid Triad** Leptin toxicity
**I**	Insulin toxicity (endogenous hyperinsulinemia-hyperproinsulinemia) IGF-1 – (GH-IGF-I axis) toxicity: This may serve to increase bone metabolism within the media of the AVW Inflammation toxicity
**G**	Glucotoxicity (compounds peripheral insulin resistance) and induces reductive stress through the sorbitol/polyol pathway Pseudohypoxia (increased NADH/NAD ratio)
**H**	Hypertension toxicity Homocysteine toxicity hs-CRP
**T**	Triglyceride toxicity: **Obesity toxicity – Lipid Triad**
**U**	Uric Acid – Xanthine Oxidase toxicity: Uric acid is an antioxidant early in physiological range of atherogenesis and a conditional prooxidant late when elevated through xanthine oxidase enzyme and generation of ROS: thus generating the paradoxical (antioxidant → prooxidant):
	**URATE REDOX SHUTTLE**
	Endothelial cell dysfunction with eNOS uncoupling, decreased eNO and increased
	**ROS**

**Table 4:** The RAAS Acronym: Redox Stress Reduction – Global risk reduction

**R**	**Reductase inhibitors **(HMG-CoA). Decreasing modified LDL-cholesterol, i.e., oxidized, acetylated LDL-cholesterol. Decreasing triglycerides and increasing HDL-cholesterol. Improving endothelial cell dysfunction. Restoring the abnormal Lipoprotein fractions. Thus, decreasing the redox and oxidative stress to the arterial vessel wall and myocardium.
**A**	AngII inhibition or receptor blockade: **ACEi-prils. ARBs-sartans. **Both inhibiting the effect of angiotensin-II locally as well as systemically. Affecting hemodynamic stress through their antihypertensive effect as well as the deleterious effects of angiotensin II on cells at the local level – decreasing the stimulus for O_2_. production. The positive effects on microalbuminuia and delaying the progression to end stage renal disease. Plus the antioxidant effects within the arterial vessel wall and capillary. Antioxidant effects. **Aspirin **antiplatelet, anti-inflammatory effect on the diabetic hyperactive platelet. **Adrenergic (non-selective blockade) **in addition to its blockade of prorenin → renin conversion. **Non dihydropyridine calcium channel blocker – antagonists **are preferred over dihydropyridine calcium channel blockers – antagonists in CKD.
**A**	**Aggressive control of diabetes **to HbA1c of less than 7. This usually requires combination therapy with the use of insulin secretagogues, insulin sensitizers (PPAR-gamma agonists), biguanides, alpha-glucosidase inhibitors, and ultimately exogenous insulin. Decreasing modified LDL cholesterol, i.e., glycated-glycoxidated LDL cholesterol. Improving endothelial cell dysfunction. Also decreasing glucotoxicity and the oxidative-redox stress to the intima and pancreatic islet. **Aggressive control of blood pressure**, which usually requires combination therapy. **Aggressive control of homocysteine **with folic acid with its associated additional positive effect on re-coupling the eNOS enzyme reaction by restoring the activity of the BH_4 _cofactor to run the eNOS reaction via a folate shuttle mechanism and once again produce eNO. **Aggressive control of uric acid **levels with xanthine oxidase inhibitors (allopurinol and oxypurinol) should be strongly considered in view of the prevailing literature in order to achieve more complete **Global Risk Reduction** **Aggressive control of hyperphosphatemia and elevated PTH **and control intake of oral calcium while limiting vitamin D. Calcium carbonate should no longer be used as a phosphate binder in patients with calciphylaxis or patients at very high risk for developing calciphylaxis.
**S**	**Statins. **Improving plaque stability (pleiotropic effects) independent of cholesterol lowering. Improving endothelial cell dysfunction. Moreover, the indirect antioxidant anti-inflammatory effects within the islet and the arterial vessel wall promoting stabilization of the unstable, vulnerable islet and the arterial vessel wall. **Style. **Lifestyle modification (weight loss, exercise, and change eating habits). **Stop Smoking.** **Statins: Emerging information: **Positive effect on the Quality of HDL-C is emerging. i.e. prevents ox HDL-C and increases HDL-C 5–10 percent.

**Table 5:** Proposed regulators of VOC

**Positive Regulators**	**Negative Regulators**
**Minerals: Ca^++ ^Pi, Phosphate/Pit-1 → Cbfa-1**	**Fetuin-A**
**Vitamin D**	
**BMP-2** Bone morphogenic protein	**MGP** Matrix gammacarboxyglutamate (Gla)-protein **Inhibits BMP-2**
**Warfarin**	
**Warfarin may also cause skin necrosis similar to CPLX via an impairment of Protein C with thrombosis in venules resulting in subcutaneous ischemia and Vitamin K – GLA inhibition.**	**GLA** gammacarboxyglutamate
**Inflammation: TNFalpha, IL-6**	**IL-10**
**Oxidized – retained – modified lipids (OxLDL-C, OxVLDL beta lipoproteins) and Low alpha lipoproteins: HDL-C**	**Osteopontin: OPN** (may be a positive and negative regulator)
**ROS** **Oxidative-Redox Stress**	**Osteoprotegerin OPG?** Elevated in human atherosclerosis and CVD yet paradoxically when you have a mouse model with knockout of OPG there is increased osteoporosis and VOC.
**A-FLIGHT TOXICITIES **(table 3)	
**Glucose**	
**Hormones: Insulin, IGF-1, Amylin, Leptin, and PTH**	
**I or II Hyperparathyroidism**	
**HYPOXIA activates hypoxia inducible factor Hif-1, ROS → Vascular angiogenesis** **Vasa Vasorum**: **Multipotential → Pericyte**	**RANK and RANKL** These are involved but as with OPG the entire story is still evolving.
**PERICYTE**	

**Table 6:** Differential diagnosis of cutaneous ulcers

**Drug reactions:**
**Warfarin**
**Circulatory Disorders:**
**Venous stasis**
**Peripheral arterial disease**
**Necrotizing vasculitis – Collagen vascular diseases -**
**Infectious endocarditis with septic emboli**
**Cholesterol emboli**
**CALCIPHYLAXIS (CPLX) – CALCIFIC UREMIC ARTERIOLOPATY (CUA)**
**Diabetes: (T1DM – T2DM)**
**Peripheral neuropathy**
**Peripheral vascular disease accelerated atherosclerosis (atheroscleropathy) ASO Insulin injection sites (sharp trauma)** **Necrobiosis lipoidica diabeticorum?**
**Trauma**
**Sharp – Blunt – pressure ulcers at pressure points due to immobilization**
**Thermal (Hot or Cold **[cryoglobulinaemia]) – **Radiation**
**Radiation therapy**
**Miscellaneous**
**Brown Recluse spider bite**
**Malignant tumors**
**Inflammatory Diseases**
**Collagen Vascular Diseases – Necrotizing vasculitis specifically SLE, systemic sclerosis (Scleroderma)** **Pyoderma gangrenosum **usually associated with chronic inflammatory diseases such as: ulcerative colitis, Crohn's disease, and rheumatoid arthritis

**Table 7:** The positive effects of eNOS and eNO

• Promotes vasodilatation of vascular smooth muscle.
• Counteracts smooth muscle cell proliferation.
• Decreases platelet adhesiveness.
• Decreases adhesiveness of the endothelial layer to monocytic WBCs (the "teflon effect").
• Anti-inflammatory effect.
• Anti-oxidant effect. It scavenges reactive oxygen species locally, and acts as a chain-breaking antioxidant to scavenge ROS.
• Anti-fibrotic effect. When NO is normal or elevated, matrix metalloproteinases (MMPs) are quiescent; conversely if NO is low, MMPs are elevated and active. MMPs are redox sensitive.
• NO inhibits prooxidant actions of uric acid during copper-mediated LDL oxidation.
• NO has diverse anti-atherosclerotic actions on the arterial vessel wall including antioxidant effects by direct scavenging of ROS – reactive nitrogen species RNS.
